# Improving carbon stock estimates in plantation forestry: Site-specific allometric equations for *Gmelina arborea* in Ghana’s Kwahu Highlands

**DOI:** 10.1371/journal.pone.0355482

**Published:** 2026-08-07

**Authors:** Benjamin Wiafe Asare, Michael Ansong, Kwadwo Boakye Boadu, Prince Owusu

**Affiliations:** 1 Department of Silviculture and Forest Management, Faculty of Renewable Natural Resources, Kwame Nkrumah University of Science and Technology, Kumasi, Ghana; 2 Department of Wood Science and Technology, Faculty of Renewable Natural Resources, Kwame Nkrumah University of Science and Technology, Kumasi, Ghana; Dev Bhoomi Uttarakhand University, INDIA

## Abstract

Accurate estimation of tree biomass and volume is essential for carbon accounting and climate reporting. This study developed site-specific allometric models for predicting stem volume and aboveground biomass of *Gmelina arborea* in the high-elevation Kwahu Highlands of Ghana. The trees were grouped into three DBH classes. DBH, total height, merchantable height, bole height, and crown dimensions of 45 trees were measured. Twenty-four of these were randomly destructively sampled for volume and biomass estimation. A range of linear, power, and log-linear regression models were evaluated to identify the most accurate predictors. Tree structural attributes changed systematically with DBH. As DBH increased, total height increases rapidly, bole height increased more gradually, and crown length showed strong growth. Expanded log-linear models [ln(Y) = a + b_1_*ln(x) + b_2_*ln(h) + b_3_*ln(p)] incorporating DBH, total height, and wood density provided the best fit for both stem volume and biomass (R^2^ > 0.90), with minimal error and strong agreement with observed values. Simpler DBH + height models showed significant deviations for biomass estimation but performed adequately for volume prediction. These findings highlight the importance of integrating multiple predictors in biomass models for unmanaged highland plantations. The resulting equations improve carbon stock estimation accuracy, supporting climate policy and sustainable forest management in tropical highland ecosystems.

## 1 Introduction

Plantation forests now cover about 131 million hectares, roughly 3% of global forest area, and that planted forest area has grown by approximately 123 million hectares since 1990 [1]. These plantations have demonstrated significant potential to increase carbon stocks [2–4] as the planted trees accumulate biomass rapidly [5,6]. Accurate carbon accounting in plantations is essential for climate policy and for meeting national and international reporting obligations, such as those under REDD+ and the Paris Agreement [7,8]. Reliable carbon accounting is particularly important in countries like Ghana where plantation forestry is expanding rapidly and forms part of national climate strategies.

In Ghana, plantation forestry has become a key strategy for restoring degraded lands, reducing pressure on natural forests, and improving rural livelihoods [9]. Decades of deforestation driven by agricultural expansion, illegal logging, and mining have left large areas of forest reserves severely degraded, with the country losing nearly 20 percent of its forest cover since 1990 [10]. To reverse this trend, Ghana launched the National Forest Plantation Development Programme and later the Forest Plantation Strategy (2016–2040), which aim to rehabilitate degraded reserves and off-reserve areas through large-scale tree planting and agroforestry interventions [11]. The Modified Taungya System has become a flagship model, allowing farmers to grow food crops alongside trees, providing short-term income while establishing long-term forest cover [12,13]. These initiatives do more than restore ecosystems; they contribute to carbon sequestration, and align with Ghana’s REDD+ strategy and commitments under the Paris Agreement [14,15].

Among the species widely cultivated in Ghana is *Gmelina arborea*, a fast-growing deciduous tree valued for its timber, fuelwood, and pulp [9]. Its popularity has grown rapidly due to its high productivity and adaptability to diverse soil types [16]. Strong growth performance under short rotations makes it a preferred species for commercial plantations and landscape restoration schemes. Importantly, *Gmelina arborea* is increasingly utilized in carbon sequestration projects because of its exceptional biomass accumulation and ability to store significant amounts of carbon. Studies in Ghana have shown that two-year-old Gmelina monocultures store approximately 28.78 Mg C ha^-1^, which is substantially higher than four-year *Tectona grandis* stands [9].

Accurately estimating aboveground biomass (AGB) and carbon stocks for *Gmelina arborea* remains a major challenge, especially in high-elevation landscapes such as the Kwahu Highlands in Ghana [9,17]. Current allometric models for this species, in the country and elsewhere, are mostly developed for lowland conditions [18–23]. [22] for example found that younger plantations, up to 15 years old, exhibited greater sequestration potential due to their rapid biomass accumulation. [18] observed that both total height and biomass of Gmelina increased with age, whereas [19] concluded that Gmelina plantations in Nigeria accumulate substantial biomass, with a significant proportion stored in the stem, indicating strong potential for timber production and carbon storage.

Applying these generalized models to montane environments often produces large errors because they fail to capture site-specific ecological factors [20,22,24]. Elevation influences nutrient availability, evapotranspiration, and competition dynamics, which shape tree growth patterns, wood density, and biomass allocation [25,26]. These variations make it essential to develop locally calibrated, species-specific models that reflect the unique conditions of highland ecosystems. Without such models, carbon accounting for plantation forestry in these areas will remain uncertain, undermining both national reporting and climate mitigation strategies.

To contribute to filling this gap, we developed a site-specific allometric model for estimating aboveground biomass of *Gmelina arborea* in the Kwahu Highlands of Ghana. Our study focused on two key objectives. First, we examined how diameter at breast height (DBH) relates to tree structural attributes and biomass components. Second, we tested different regression models; linear, and non-linear, to identify the most accurate function for predicting volume and biomass. We based the model on destructive sampling and incorporated locally measured wood density values to ensure it reflects the ecological realities of high-elevation environments. By producing an empirically derived equation tailored to these conditions, we aim to improve carbon stock estimation in plantation forestry and strengthen Ghana’s capacity for climate reporting and sustainable forest management. More broadly, the findings contribute to the growing body of research on site-specific allometry and highlight the importance of ecological context in tropical carbon accounting.

## 2 Materials and methods

### 2.1 Study site

**Fig 1:**
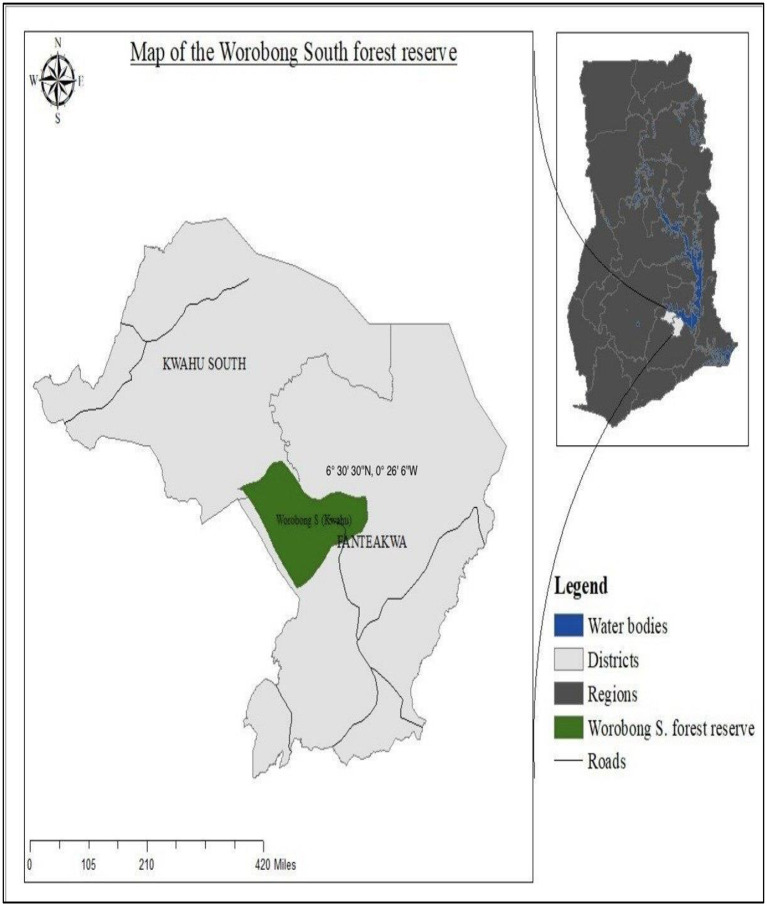
Map of study areas. Forest reserve boundaries were obtained from [27]. The map was produced by authors with QGIS software.

This study was conducted in the Kwahu portion of the Worobong South Forest Reserve (WSFR), located in Ghana’s Eastern Region (Fig 1). The site lies at approximately 6°30′30″N latitude and 0°26′6″W longitude, within the administrative boundaries of the Kwahu South District and managed under the Mpraeso Forest District. The reserve is bordered by the Akim portion of the Worobong South Forest Reserve to the west and the Southern Scarp Forest Reserve to the south.

Fieldwork took place in Compartment 33 of the reserve, which forms part of a broader landscape covering about 11,453 hectares. The area falls within Ghana’s Moist Semi-Deciduous Forest Zone, a region known for its moderate to high rainfall (1,250–1,500 mm annually) and warm temperatures, with daily highs ranging from 27°C to 35°C and lows between 19°C and 23°C. The terrain sits at an average elevation of 432 meters above sea level and is underlain by forest ochrosol soils, which support a variety of forest vegetation.

Notable water bodies within the reserve include the Mia and Oworobong rivers, which contribute to the area’s ecological richness. Surrounding communities such as Miaso, Pillar 10, and Kwaku Wutu are closely connected to the forest, both geographically and economically.

The specific study site is a degraded section of the forest that has been converted into a *Gmelina arborea* plantation. The stand is characterized by dense undergrowth, scattered saplings, and a generally poor structural form. Notably, no silvicultural treatments have been applied, resulting in an unmanaged and uneven forest structure. This setting provided a realistic context for assessing biomass accumulation under typical plantation conditions in Ghana’s highland forests. Fieldwork for this study was conducted with official approval from the Forestry Commission of Ghana, through the Mpraeso Forest Services Division, which manages the study area. Access to the forest reserve and the concession area was authorized prior to data collection, permitting field measurements.

### 2.2 Sample collection and processing

*Gmelina arborea* was selected because it is one of the principal plantation species in Ghana, yet species-specific stem volume and aboveground biomass equations are lacking for high-elevation plantations in the Kwahu landscape. Since allometric relationships are species-specific, the development of reliable prediction models requires calibration using measurements from a single species under local site conditions. Tree selection for this study was guided by the operational harvesting plan of the plantation, in accordance with Ghana’s forestry regulations. Hence, only trees with DBH ≥ 20 cm that were scheduled for harvesting were eligible for destructive sampling. To ensure an unbiased and representative sample, a simple random sampling method was used. Random numbers were generated using Microsoft Excel, and these were matched to the tagged trees in the field. This approach allowed for an objective selection process, free from researcher bias. To account for variation in tree size, the sampled population was stratified into three diameter at breast height (DBH) classes: 20–30 cm, 31–40 cm, and ≥41 cm to ensure balanced representation of small, medium, and large harvestable trees while maintaining an adequate number of observations within each class for model development. Increasing the number of DBH classes would have reduced the number of trees available per class without providing substantial additional information, given the total sample size. From each class, 15 trees were randomly selected, yielding a total sample of 45 trees with DBH values ranging from 25.0 cm to 56.7 cm. For all selected trees, measurements were taken for DBH (cm), total height (m), merchantable height (m), bole height (m), crown length (m), and crown diameter (m).

**Plate 1:**
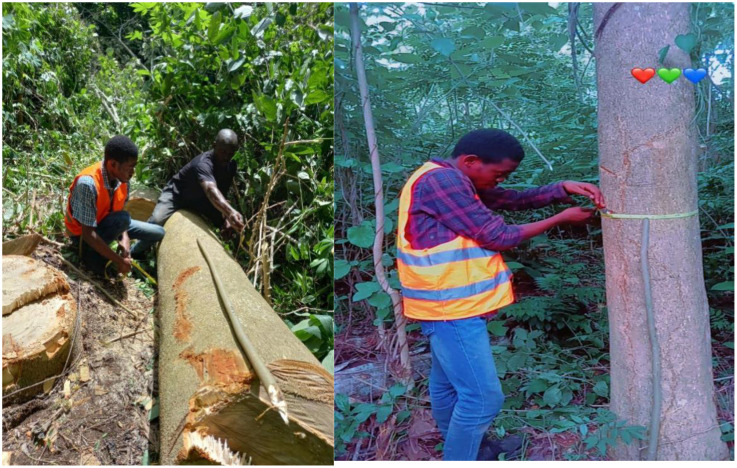
Sample of tree measurement.

From this pool, 24 trees (eight per DBH class) were randomly chosen and felled using a chainsaw for destructive sampling (Plate 1). Destructive sampling was employed in this study because it provides the most reliable and direct method for quantifying tree biomass and developing species-specific allometric equations [28]. While non-destructive approaches such as remote sensing or the use of existing allometric models are available, they rely on prior calibrations that may not accurately represent local site conditions, species characteristics, or stand structure. Given the objective of this study to develop site-specific biomass and volume equations for *Gmelina arborea* in the Kwahu highlands, destructive sampling was necessary to obtain precise measurements of tree components and minimize estimation bias. This approach is widely recognized as the standard method for calibrating allometric models in forest biomass studies. Of the 24 trees, 19 trees were used to develop allometric models for predicting stem volume and total aboveground biomass, while the remaining five were reserved for model validation. [28] recommended that, when using a destructive approach to estimate woody biomass, sampling 17–95 trees is appropriate.

Once felled, each tree was separated into its main components, stem and branches. Leaf biomass was excluded from the analysis because it contributes a relatively small proportion to total aboveground biomass in *Gmelina arborea* (~3%) [19]. In addition, leaf biomass is highly variable seasonal turnover, making it less reliable for developing stable allometric relationships.

To estimate the volume of each tree, we measured stem diameters every 2.0 meters and branch diameters every 1.0 meter, starting from the base of each section. These measurements followed the guidelines of [29] for trees with a DBH greater than 20 cm. Using these data, we calculated green volume with Smalian’s formula, a widely used approach for estimating the volume of irregularly shaped logs and branches.


$$\begin{array}{c} V=𝛴\frac{(Bi+bi)L}{\text{2}} \end{array}$$

Where,

V = volume using Smalian’s formula,

Bi = cross-sectional area of the bigger end of the bole,

bi = cross-sectional area of the smaller end of the bole,

L = length of the tree.

We used a chainsaw to collect sample discs from both the trunks and branches of randomly selected tree. Wood density samples were obtained from three representative trees, one from each DBH class, to capture size-related variation. From the trunk, two discs were taken; one at the butt end and another near the top. For the branches, we randomly selected one large branch (≥2 cm diameter) and one small branch (<2 cm diameter) and a disc was removed from each, yielding a total of twelve (12) subsamples.. To prevent moisture loss, all samples were sealed in polythene bags and transported to the Natural Resources Management General Laboratory for analysis of wood basic density and volume.

In the lab, we prepared rectangular subsamples from the trunk and branch discs for volume determination. These were oven-dried at 105 °C until they reached a constant weight, following the procedure described by [29]. Wood basic density for trunks and branches was then calculated using the formula


$$\begin{array}{c} WD=\frac{M}{V} \end{array}$$

Where,

WD = wood density

M = mass

V = volume

The wood density was used to get the biomass of the trunk and branches as follows


$$\begin{array}{c} \text{Trunk biomass }(\text{TM})=\text{wood basic density}*\text{trunk volume} \end{array}$$

$$\begin{array}{c} \text{Branch biomass }(\text{BM})=\text{wood basic density}*\text{branch volume} \end{array}$$

The total aboveground biomass of each tree was calculated as


$$\begin{array}{c} \text{AGB}=\text{TTM}+\text{TBM} \end{array}$$

Where, AGB = Aboveground biomass


$$\begin{array}{c} \text{TTM}=\text{Total mass of trunk }/\text{ total trunk biomass} \end{array}$$

$$\begin{array}{c} \text{TBM}=\text{Total mass of branch }/\text{ total branch biomass} \end{array}$$

### 2.3 Statistical analysis (model fitting)

The simple power model (Table 1) was used to determine how well DBH predicts Total Height, Merchantable Height, Bole Height, Crown Length, and Crown Diameter. This analysis supports forest inventory modeling and silviculture decisions by identifying which attributes are most strongly correlated with DBH. For each regression, a best-fit line was computed, 95% confidence intervals were plotted around the regression line, and the regression equation and coefficient of determination (R^2^) were annotated on each plot.

**Table 1:** Generic biomass model used to assess stem volume and total above ground biomass.

Input Level	Model Type	Equation	Equation number
DBH only	Linear	Y = a + b*x	1
DBH only	Power	Y = a*xᵇ	2
DBH only	Log-linear	ln(Y) = a + b*ln(x)	3
DBH + Height	Linear	Y = a + b_1_*x + b_2_*h	4
DBH + Height	Power	Y = a·(x^2^·h)ᵇ	5
DBH + Height	Log-linear	ln(Y) = a + b*ln(x^2^*h)	6
DBH + Height + Density	Linear	Y = a + b_1_*x + b_2_*h + b_3_*p	7
DBH + Height + Density	Power	Y = a*(p*x^2^*h)ᵇ	8
DBH + Height + Density	Log-linear	ln(Y) = a + b*ln(p*x^2^*h)	9
DBH + Height + Density	Expanded log-linear	ln(Y) = a + b_1_*ln(x) + b_2_*ln(h) + b_3_*ln(p)	10

Note: x is DBH; h is Total Height; p is Wood density; Y is either Stem volume or Total above ground biomass; a, b_1_, b_2_, and b_3_ are constants.

Various model forms, including linear, power, and log-linear, were evaluated to develop predictive models for estimating stem volume (m^3^) and total above-ground biomass using combinations of DBH, height, and wood density (Table 1). Model performance was evaluated using R^2^ or pseudo-R^2^, Mean Absolute Percentage Error (MAPE), and Akaike Information Criterion (AIC). Residual plots were used to assess model assumptions. The analysis was conducted using the R programming language.

Prior to the regression analysis, the assumptions of OLS were verified. Pairwise correlation analysis was conducted to examine the relationships among explanatory variables and to assess potential multicollinearity. The correlation coefficients were within acceptable limits, indicating no severe multicollinearity. This was further confirmed using Variance Inflation Factors (VIF). In addition, residual normality was assessed using Q–Q plots and the Shapiro–Wilk test, while homoskedasticity was assessed using a plot of standardized residuals against standardized predicted values, which showed no systematic pattern.

The selected best model(s) outputs for both volume and biomass were compared against each other and against the calculated values from the five trees reserved for model validation to evaluate the validity and consistency of different models. Each comparison was analysed using a paired t-test, with a significance level of p < 0.05 used to assess statistical differences. The standard error of the mean (SEM), t-statistic, and p-values were calculated for each comparison.

## 3 Results

### 3.1 Descriptive statistics for selected trees

DBH ranges from 25.00 cm to 56.70 cm, while total height had a mean of 17.19 ± 1.27 m (Table 2). Stem/bole height and merchantable height show more consistency with means of 6.92 ± 0.21m and 6.19 ± 0.34m, respectively, and relatively low standard errors, suggesting uniformity in usable stem portions. Crown Length and Crown Diameter exhibited greater variability, with Crown Length ranging from 2.29 m to 27.41 m and Crown Diameter from 5.90 m to 16.70 m (Table 2).

**Table 2:** Descriptive statistics for sampled trees.

Variable	N	Minimum	Maximum	Mean	Standard Error
DBH (cm)	45	25.0	56.7	36.27	1.31
Total Height (m)	45	7.33	39.13	17.19	1.27
Stem/bole Height (m)	45	3.4	12.92	6.92	0.34
Merchantable Height (m)	45	3.4	9.16	6.19	0.21
Crown Length (m)	45	2.29	27.41	10.27	0.99
Crown Diameter (m)	45	5.9	16.7	10.77	0.47
Stem volume (m^3^)	24	0.237	1.645	0.757	0.01
Branch volume (m^3^)	24	0.058	0.973	0.380	0.06
Total volume (m^3^)	24	0.300	2.475	1.137	0.16
Stem biomass (Mg/tree)	24	0.107	0.740	0.341	0.04
Branch biomass (Mg/tree)	24	0.026	0.438	0.171	0.03
Total above biomass (Mg/tree)	24	0.135	1.114	0.512	0.07
Wood density (g/cm^3^)	12	0.43	0.48	0.45	0.004

**Note:** Tree measurements were obtained from 45 individuals; 24 were felled for biomass and volume estimation (19 were used for model development and 5 for validation).

To get the volume and aboveground biomass, the study took respective measurements from different plant parts (branches and stems) as detailed in Table 2. The mean for stem volume and total aboveground biomass were 0.757 ± 0.01 (m^3^) and 0.512 ± 0.07 (Mg/tree), respectively. Stem volume varied between 0.237 and 1.645 m^3^/tree, while total tree volume varied from 0.300 to 2.475 m^3^/tree (Table 2). Similarly, the total aboveground biomass ranges from 0.135 to 1.114 Mg/tree (Table 2). The stem accumulated greater biomass compared to its branches, with 66.59% of the aboveground biomass in the former and 33.41% in the latter. For the purpose of biomass modelling, an average wood density value of 0.45 g cm^-3^ was used to represent *Gmelina arborea* in the study area.

### 3.2 Relationship between DBH and key tree attributes

**Fig 2:**
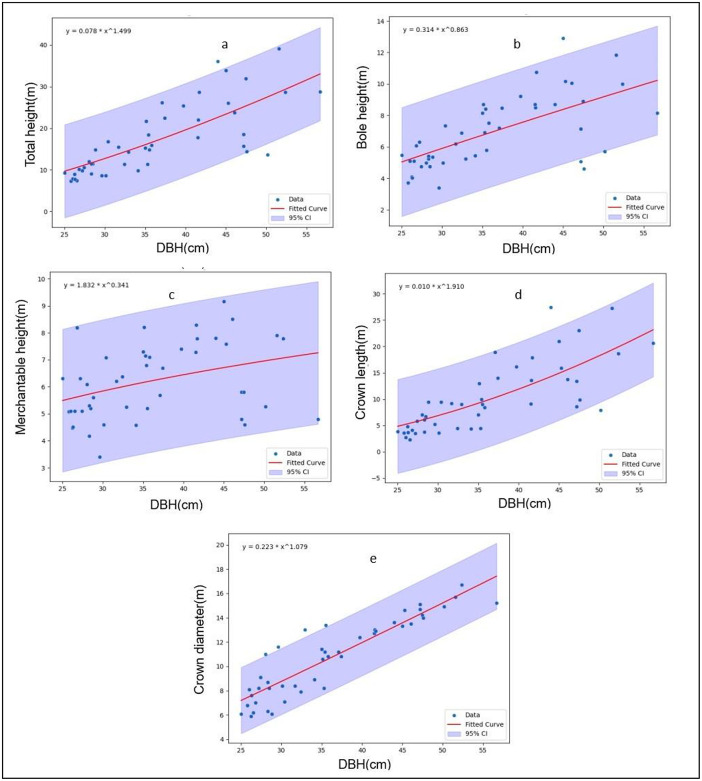
Relationship between DBH, Total height (a), Bole height (b), Merchantable height(c), Crown lenght (d), and Crown diameter (e). Power model (Y = a (X)^b^) was used to fit the relationship. The x-axis represents diameter at breast height (DBH, cm), while the y-axis represents the corresponding tree structural attribute in each panel. The solid red line represents the fitted power regression model, and the shaded blue region indicates the 95% confidence interval around the fitted regression line.

The power model effectively described a positive relationship between tree diameter (DBH) and all the parameters (Table 3 and Fig 2). With total height, the fitted model showed a positive relationship, explaining approximately 59% of the variation in height. The exponent *b* greater than 1 suggests that height increases more rapidly with DBH, especially in smaller trees. Bole height increased at a slower rate with DBH (coefficient = 0.3139, exponent = 0.8628, 𝑅^2^ = 44%).

**Table 3:** Models developed to evaluate the relationship between DBH, Total height, Bole height, Merchantable height, Crown length, and Crown diameter. Power model (Y = a (X)^b^) was used to obtain the coefficient, a and b values, along with the R^2^.

Variable	a	b	R-squared
Total height(m)	0.0779	1.4986	0.5867
Bole/stem height(m)	0.3139	0.8628	0.4368
Merchantable height(m)	1.8318	0.3411	0.1378
Crown length(m)	0.0104	1.9096	0.5713
Crown diameter(m)	0.2234	1.0788	0.8212

The model for merchantable height showed a weak fit, with a low explanatory power (𝑅^2^ = 14%), suggesting that DBH alone may not be a strong predictor of merchantable height. In contrast, crown length had a stronger relationship with DBH (coefficient = 0.0104, exponent = 1.9096, 𝑅^2^ = 0.57%), indicating a steep increase in crown length as DBH increases. The strongest model fit was observed for crown diameter, with a coefficient of 0.2234 and an exponent of 1.0788, explaining 82% of the variation (Table 3, Fig 2).

### 3.3 Model for predicting the stem volume of *Gmelina arborea*

We tested ten different models to estimate tree stem volume using DBH, total height, and wood density. The model that performed best was the expanded log-linear model, which included all three variables (DBH, height, and wood density). It explained more than 90% of the variation in stem volume (Table 4), had the lowest prediction error (MAPE = 11.2%), and was the most efficient in terms of model fit (AIC = –68.90). This shows that combining these three measurements provides the most accurate and reliable volume estimates.

**Table 4:** Allometric equations for volume estimation in *Gmelina arborea*. Details of the Regression prediction model, coefficient of determination (R^2^), Mean Absolute Percentage Error (MAPE), and Akaike Information Criterion (AIC) are provided.

Model	Equation	R²(%)	MAPE (%)	AIC	ΔAIC	Ranked
Expanded Log-linear Full	V = exp (5579 + 2.1233 ln(x) + 0.4463 ln(h) + 6998 ln(p))	90.8	11.2	−68.9	0.0	1
Linear DBH + Height	V = −1.01 + 0.0405x + 0.0176h	89.7	13.9	−68.7	0.2	2
Linear Full	V = 8622 + 0.0405x + 0.0176h + -19163p	89.7	13.9	−66.7	2.2	3
Power DBH + Height	V = 0.0007(x^2^ h)^0.7003^	85.5	16.4	−64.2	4.7	4
Log-linear DBH + Height	V = exp (−7.3168 + 0.7003 ln(x^2^h))	85.5	16.4	−64.2	4.7	5
Power Full	V = 0.0012 (px^2^h)^0.7003^	85.5	16.4	−64.2	4.7	6
Log-linear Full	V = exp (−6.7577 + 0.7003 ln(px^2^h))	85.5	16.4	−64.2	4.7	7
Linear DBH only	V = −1.1036 + 0.0507x	84.4	16.5	−62.9	6.0	8
Power DBH only	V = 0.00001x^2.4096^	81.9	19.0	−60.1	8.8	9
Log-linear DBH only	V = exp(−9.0273 + 2.4096 ln(x))	81.9	19.0	−60.1	8.8	10

V is stem volume; h is height; x is DBH and p is wood density.

The linear model that used just DBH and height also performed well (R^2^ = 89.7, MAPE = 13.93%, AIC = –68.70). Interestingly, adding wood density to this model, creating the full linear version, did not improve its performance, suggesting that in a linear form, wood density may not add much predictive value beyond what DBH and height already provide.

The power models followed a similar pattern. A model using only DBH explained about 81% of the variation in volume (R^2^ = 81.90) but had higher prediction error (MAPE = 19.0%) and a relatively poorer fit (AIC = –60.10). Adding height improved performance (R^2^ = 85.5, MAPE = 16.4%, AIC = –64.2), though adding density made little difference.

Interestingly, the log-linear models produced exactly the same results as the power models. This outcome is expected because power relationships become linear when variables are analysed on logarithmic scales. As a result, fitting a log-linear regression to log-transformed data is mathematically equivalent to fitting a power model to the original data, which can lead to identical parameter estimates and model performance. This equivalence between power functions and log-transformed linear models is well documented in regression literature [30,31]. Based on the AIC difference, the expanded Log-linear Full and linear with DBH and Height are the preferred models for estimating stem volume.

### 3.4 Model for predicting the aboveground biomass of *Gmelina arborea*

We tested ten different models to estimate aboveground biomass using DBH, total height, and wood density. The model that performed best was the expanded log-linear model, which included all three variables (DBH, height, and wood density). It explained more than 90% of the variation in aboveground biomass (Table 5), had the lowest prediction error (MAPE = 11.5%), and was the most efficient in terms of model fit (AIC = −80.8). Just like observed in the volume equation, combining these three measurements provides the most accurate and reliable aboveground biomass estimates of *G. arborea*. The linear model that used just DBH and height also performed well (R^2^ = 88.7%, MAPE = 17.1%, AIC = –78.4), followed by the full linear model (Table 5). The ranking of the remaining models is presented in Table 5. In terms of model selection, the result indicates that expanded log-linear model is the only suitable model, with the remaining having ΔAIC difference greater than 2.

**Table 5:** Allometric equations for aboveground biomass estimation in *Gmelina arborea*. Details of the Regression prediction model, coefficient of determination (R^2^), Mean Absolute Percentage Error (MAPE), and Akaike Information Criterion (AIC) are provided.

Model	Equation	R²(%)	MAPE (%)	AIC	ΔAIC	Ranked
Expanded Log-Linear Full	Y = exp(−4285 + 2.4503 * ln(x) + 0.4536 * ln(h) + −5352 * ln(p))	91.2	11.5	−80.8	0.0	1
Linear DBH + Height	Y = −0.8073 + 0.0315 * x + 0.0109 * h	88.8	17.1	−78.4	2.4	2
Linear Full Model	Y = −14371 + 0.0315 * x + 0.0109 * h + 31934* p	88.8	17.1	−76.4	4.4	3
Linear DBH	Y = −0.8653 + 0.0378 * x	85.1	17.3	−75.0	5.8	4
Power Full Model	Y = 0.0004 * (p * x^2^ * h)^0.7617^	83.6	19.9	−73.2	7.6	5
Log-Linear Full Model	Y = exp(−7.7147 + 0.7617 * ln(p * x^2^ * h))	83.6	19.9	−73.2	7.6	6
Power DBH + Height	Y = 0.0002 * (x^2^ * h)^0.7617^	83.6	19.9	−73.2	7.6	7
Log-Linear DBH + Height	Y = exp(−8.3229 + 0.7617 * ln(x^2^ * h))	83.6	19.9	−73.2	7.6	8
Log-Linear DBH	Y = exp(−10.2895 + 2.6506 * ln(x))	83.3	17.6	−72.8	8.0	9
Power DBH	Y = 0.00001 × x^2.6506^	83.3	17.6	−72.8	8.0	10

Y is above ground biomass; h is height; x is dbh and p is wood density.

### 3.5 Model validation

#### 3.5.1 *Volume.*

We compared the performance of the best model (Expanded Log-linear Full), the second-best model (Linear DBH + Height), and the calculated values derived from the validation dataset. The results indicate that the estimates from the best model were not significantly different from the calculated values (Table 6). Similarly, no significant differences were observed between the calculated values and the second-best model, nor between the best and second-best models. These findings suggest that both models provide comparable volume estimates that align closely with the observed data.

**Table 6:** Paired t-test results for model validation.

Parameter	Model comparison	n	Mean Difference	SEM	t(4)	p-value
Volume	A and B	5	0.316	0.120	2.63	0.058
	B and C	5	0.034	0.052	0.67	0.541
	A and C	5	0.350	0.128	2.73	0.053
Biomass	D and E	5	0.098	0.038	2.56	0.063
	D and F	5	−0.319	0.094	−3.36	0.028

Note: **A** is V = exp(5579 + 2.1233 * ln(x) + 0.4463 * ln(h) + 6998 * ln(p)); **B** is calculated volume from field dataset for validation; **C** is V = −1.01 + 0.0405 * x + 0.0176 * h; **D** is calculated biomass from field dataset for validation; **E** is Bi = exp(−4285 + 2.4503 * ln(x) + 0.4536 * ln(h) + −5352 * ln(p)); **F** is Bi = −0.8073 + 0.0315 * x + 0.0109 * h;, h is height; x is dbh, p is wood density, V is stem volume; Bi is total above ground biomass.

#### 3.5.2 *Biomass.*

For biomass estimation, we evaluated the best model (Expanded Log-linear Full), the second-best model (Linear DBH + Height), and the calculated values. The results showed no significant differences between the calculated values and the estimates from the best model. However, a significant difference was found between the calculated values and the estimates from the second-best model (Table 6), indicating that the second-best model may be less accurate for biomass prediction in this context.

## 4 Discussions

### 4.1 Characteristics of selected trees

The growth and biomass characteristics of *Gmelina arborea* in this study showed notable variability among individual trees (Table 2), reflecting site conditions and the absence of silvicultural management in the Kwahu highlands of Ghana. The mean DBH (36.27 ± 1.31 cm) and height (17.19 ± 1.27 m) indicate moderately developed trees under semi-natural conditions. These values are close to those reported in managed plantations in Nigeria [32], although the slightly lower height observed here is likely linked to the high elevation and cooler microclimate that limit vertical growth. Similar findings in Indonesia [33] reinforce the role of local climate in shaping tree form.

The mean stem volume (0.757 ± 0.01 m^3^) was lower than the 0.96 ± 0.61 m^3^ reported by [32] at similar DBH, probably due to differences in stand management and site productivity. The wide gap between total and merchantable height (11 m) contrasts sharply with the 3.18 m reported by [34], indicating that a large proportion of the height is unmerchantable, likely due to the stand being unmanaged. [35,36] noted that larger crowns signify vigorous canopy development and efficient light absorption. Thus, despite lower height growth, *Gmelina arborea* maintains strong photosynthetic potential under open-grown, highland conditions.

The wood density (0.45 g cm^-3^) aligns with the range given in the world wood density database (0.39 to 0.48) [37], suggesting stability of this trait across ecological zones. We found that biomass partitioning in the study site was 66.6% in stems and 33.4% in branches. While stems still accounted for the majority of biomass, the difference between stem and branch allocation was smaller than reported in previous studies, which often found more extreme stem dominance. For example, [19] reported 84% stem and only 13% branch biomass in managed plantations in southwestern Nigeria, while [38] observed 71% stem and 22.9% branch biomass in Bangladesh. Our results likely reflect the influence of elevation. Trees growing at higher altitudes often adapt structurally to cope with wind exposure and lower temperatures [39,40]. These conditions can promote greater branch development for mechanical stability and light interception, producing a biomass allocation pattern that differs from lowland stands [40]. Overall, species at higher elevations tend to allocate relatively more biomass to branches than stems, contrasting with the stem-dominated pattern common in lowland plantations [39]. This evidence suggests that elevation-driven ecological pressures play a critical role in shaping carbon allocation strategies in trees.

### 4.2 Relationship between DBH and key tree attributes

The power model results revealed consistently positive allometric relationships between diameter at breast height (DBH) and all measured structural attributes of *Gmelina arborea* (including total height, bole height, merchantable height, crown length, and crown diameter) (Table 3 and Fig 2). This confirms that DBH is a strong predictor of overall tree size and architecture. The strength of these relationships varied, with crown diameter (R^2^ = 0.82) and total height (R^2^ = 0.59) showing the best fits, whereas merchantable height exhibited the weakest correlation (R^2^ = 0.14). These results collectively indicate that as stem diameter increases, trees tend to expand substantially in crown dimensions and total height, although merchantable height is more sensitive to site and form factors such as branching pattern, slope, and past disturbance.

The relationship between DBH and total height followed a positive power function (a = 0.0779, b = 1.4986, R^2^ = 0.59), demonstrating that tree height increases rapidly with diameter growth, particularly among smaller individuals. This finding is consistent with earlier results from [41], who reported strong DBH–height coupling in *Gmelina arborea* plantations in Central India. The high exponent (b > 1) observed in the present study suggests more accelerated vertical growth, possibly due to competition for light in the uneven stands of the Kwahu highlands. The model for bole height (a = 0.3139, b = 0.8628, R^2^ = 0.44) indicated that diameter expansion outpaced height increment. The exponent below 1 reflects a sub-proportional relationship where trees increase in girth faster than they elongate, a trend typical of maturing stands. Similar findings were reported by [42], where diameter continued to increase even after height stabilized. The weak DBH–merchantable height relationship (R^2^ = 0.14) underscores that diameter alone is a poor predictor of merchantable height under unmanaged conditions. This aligns with [43], who noted large variability in merchantable height among Gmelina trees in Nigerian plantations due to irregular branching and stem forking. In the highlands, where no silvicultural operations have been applied, trees form irregular boles, resulting in substantial variation in merchantable sections and overall stem form.

In contrast, the relationships between DBH and crown attributes were notably stronger. Crown length exhibited a steep positive scaling (a = 0.2405, b = 1.9096, R^2^ = 0.57), implying that larger diameters correspond to disproportionately larger and longer crowns. This suggests that as trees expand in diameter, they invest heavily in crown development, likely to maximize photosynthetic capacity and light interception. [44] observed a similar pattern, emphasizing that crown expansion reflects both genetic potential and environmental opportunity. The wide variation in crown lengths (2.29–27.41 m) observed in this study reflects the uneven terrain and spatial heterogeneity of the highlands, where canopy openings caused by slope or wind exposure promote asymmetric crown development. The DBH–crown diameter relationship was the strongest among all measured parameters (a = 0.2234, b = 1.0788, R^2^ = 0.82), confirming that stem diameter is an excellent predictor of canopy spread. This aligns with findings by [34], who similarly reported that DBH explained over 80% of crown diameter variation in *Gmelina arborea* and other tropical species. Such strong coupling between DBH and crown dimensions is expected in open-grown or unmanaged stands, where competition is minimal, and trees allocate growth resources to horizontal expansion for enhanced light capture and mechanical stability.

### 4.3 Stem volume models

The performance of the ten regression models tested for predicting the stem volume of *Gmelina arborea* in the Kwahu highlands revealed that the expanded log-linear model incorporating DBH, total height, and wood density produced the best fit. This model explained over 90% of the variation in stem volume, with the lowest Mean Absolute Percentage Error (MAPE = 11.2%) and the most efficient Akaike Information Criterion (AIC = –68.90). The second-best model was the linear model using DBH and height (R^2^ = 89.7%, MAPE = 13.93%, AIC = –68.70), while the inclusion of wood density in this linear form did not enhance predictive strength (Table 4).

These results are consistent with numerous studies that have reported DBH and total height as the most influential variables in tree volume estimation. For instance, [41] in India observed that models using both DBH and height produced better predictions of *Gmelina arborea* stem and total volume than those using DBH alone. Similarly, [43,45] in Nigeria demonstrated that incorporating height with DBH improves model accuracy, as height captures site productivity and vertical growth potential, complementing the diameter’s reflection of basal expansion. The present study confirms this trend but further shows that including wood density enhances predictive accuracy when used in logarithmic form, highlighting the role of wood properties in refining model precision, particularly across variable ecological conditions.

The superior performance of the expanded log-linear model also aligns with findings by [46], who tested several non-linear regression forms for *Gmelina arborea* in Nigeria and recommended that log-linear models are good for predicting the volume of Gmelina. [32] found in their study on *Gmelina arborea* plantations in southeastern Nigeria that logarithmic equations provided the most accurate and practical volume estimations, outperforming linear, power, exponential, and polynomial forms.

The fact that adding wood density improved model performance in the expanded log-linear model, but not in the linear or power forms, can be attributed to the way density interacts multiplicatively with DBH and height under logarithmic transformation. In highland environments such as the Kwahu highlands, where soil depth, texture, and moisture availability vary considerably, wood density can reflect site-induced variations in growth efficiency and carbon allocation. The enhanced accuracy of the log-linear model thus suggests that wood density acts as an ecological modifier, stabilizing predictions under heterogeneous conditions.

The success of the expanded log-linear model under these conditions suggests that it is well-suited for modelling naturally regenerated or unmanaged stands where structural variability is high. Conversely, the linear and power models perform well in even-aged managed plantations, as shown by [32,41] respectfully, where stand structure is more uniform.

These results indicate that both the Expanded Log-linear Full model and the Linear DBH + Height model produce volume estimates that are statistically indistinguishable from the observed (calculated) values. The performance of the best model (Expanded Log-linear Full), the second-best model (Linear DBH + Height), and the calculated values derived from the validation dataset were compared, and there were no significant differences (Table 6). The absence of significant differences across all comparisons implies that the two models perform similarly well and provide reliable estimates of stem volume for *Gmelina arborea* in the study area, although the Expanded Log-linear Full model ranked highest based on statistical fit criteria.

### 4.4 Aboveground biomass estimation models

The results of this study demonstrated that the expanded log-linear model incorporating diameter at breast height (DBH), total height, and wood density provided the most reliable estimate of aboveground biomass (AGB) for *Gmelina arborea* in the Kwahu highlands. This model explained over 90% of the variation in AGB (R^2^ = 91.2) and yielded the lowest mean absolute percentage error (MAPE = 11.5%) and Akaike Information Criterion (AIC = –80.8). The strong performance of this model highlights the advantage of combining structural and functional tree attributes when developing local allometric equations. Similar observations have been made by [47], who emphasized that including both total tree height and wood density substantially improves model precision and reduces estimation bias across tropical forests. The relatively high predictive power of the combined model compared with the DBH-only and DBH–height models align with findings by [48], who reported similar improvements when additional structural variables were included. [29] likewise noted that incorporating total height and wood density improves model performance.

The superiority of the expanded log-linear model reinforces the need for context-specific allometric equations. While pantropical models such as those proposed by [47] offer broad applicability, local models reflect species behaviour, stand structure, and environmental heterogeneity more accurately. For example, [32] identified a logarithmic model as the best predictor of *Gmelina* AGB in Edondon, Nigeria, while [49] found that a power function provided the best fit for the species under different site conditions. These contrasts highlight the influence of geography, stand history, and species architecture on allometric performance. The expanded model’s high explanatory power (>90%) indicates that DBH, height, and wood density remain reliable predictors even under unmanaged and highland conditions. This supports the argument by [50] that allometric relationships remain stable across diverse ecological contexts when appropriate structural and functional variables are included.

The validation results for aboveground biomass estimation demonstrate that the Expanded Log-linear Full model performs reliably, as its predictions did not differ significantly from the calculated biomass values. The lack of significant differences confirms that the best local model provide equivalent and accurate biomass estimates for *Gmelina arborea*. In contrast, the significant difference observed between the calculated values and the predictions from the second-best model (Linear DBH + Height) suggests that this simpler model does not adequately capture the biomass variability in the study area. Its reduced accuracy may result from the exclusion of wood density, an important functional trait influencing biomass, particularly in heterogeneous natural stands. It may also be due to the limited validation sample size. This finding highlights the importance of incorporating both structural (DBH, height) and functional (wood density) variables when developing biomass models for complex, unmanaged ecosystems. Consequently, while the second-best model may remain useful for rapid assessments, it is not recommended for precise biomass estimation in this context.

Unlike previous studies on *Gmelina arborea*, which have largely focused on lowland plantations, managed stands, or different ecological regions, this study provides locally calibrated stem volume and aboveground biomass models for an unmanaged high-elevation plantation environment in the Kwahu landscape. The study further contributes by demonstrating the performance of combined structural and functional predictors, including DBH, total height, and wood density, under conditions where tree form is influenced by limited silvicultural intervention and site-related constraints. While previous studies have developed species-specific equations for *Gmelina arborea* in other regions, the present study highlights the importance of local calibration by showing that plantation management history and environmental conditions can influence tree architecture and biomass allocation. Therefore, the developed equations provide additional evidence that species-specific models should consider local growing conditions rather than relying solely on generalized equations.

## 5 Conclusion

This study aimed to develop accurate, site-specific allometric equations for estimating stem volume and aboveground biomass of *Gmelina arborea* in the highland environment of the Kwahu landscape. By combining destructive sampling with structural measurements, the study evaluated a range of linear, power, and log-linear models and identified the predictors that best explain variation in tree volume and biomass under unmanaged high-elevation conditions.

The results showed that the expanded log-linear models incorporating DBH, total height, and wood density provided the most reliable estimates for both stem volume and aboveground biomass. These models achieved the highest explanatory power (R^2^ > 0.90), the lowest error metrics, and strong statistical agreement with observed values. For biomass estimation, the best local model performed showed no significant differences between its prediction and the calculated values. In contrast, the simpler DBH + Height biomass model showed significant deviations, indicating reduced suitability for this ecosystem. For volume estimation, both the best and second-best models showed no significant differences from observed values, suggesting that simpler models may remain useful for rapid volume assessments.

The structural characteristics of the sampled trees highlight the influence of elevation, low stand density, and absence of silvicultural management on tree architecture. These site effects reinforce the need for locally calibrated allometries, as generic or lowland-derived models may not fully capture the biomass dynamics of *Gmelina arborea* in high-elevation, unmanaged stands.

The models developed in this study are therefore most applicable to *Gmelina arborea* growing under similar ecological and management conditions in the Kwahu highlands and landscapes with similar characteristics. It is recommended that the allometric equations developed in this study be adopted for biomass and stem volume estimation in *Gmelina arborea* plantations under ecological conditions similar to those of the Kwahu Highlands. Their application can improve the accuracy of plantation inventories, carbon stock assessments, and climate reporting, thereby supporting evidence-based plantation management and carbon accounting. Beyond Ghana, these locally calibrated allometric equations provide a valuable reference for improving biomass and carbon stock estimation of *Gmelina arborea* in other tropical highland regions where similar ecological conditions, stand structures, and management limitations exist, contributing to more reliable forest monitoring, climate mitigation assessments, and carbon accounting initiatives at broader regional scales.

The validation sample size (*n* = 5) is small, so the predictive performance should be interpreted cautiously. Cross-validation would provide a more robust assessment for small datasets. Despite this limitation, the consistent model behaviour suggests stable results, but further validation with larger samples is recommended. Additional studies across age classes, stand densities, and management regimes would further strengthen the general applicability of these models.
